# Role of glomerular filtration rate‐modifying drugs in the development of anticoagulant‐related nephropathy

**DOI:** 10.14814/phy2.14697

**Published:** 2021-01-11

**Authors:** Ajay K. Medipally, Min Xiao, Brad H. Rovin, Anjali A. Satoskar, Iouri Ivanov, Shahzeb Qaisar, Sergey V. Brodsky

**Affiliations:** ^1^ Department of Pathology The Ohio State University Wexner Medical Center Columbus Ohio USA; ^2^ Department of Medicine The Ohio State University Wexner Medical Center Columbus Ohio USA

**Keywords:** 5/6 nephrectomy, anticoagulant‐related nephropathy, glomerular filtration rate

## Abstract

**Introduction:**

Anticoagulant‐related nephropathy (ARN), that was described in humans first as warfarin‐related nephropathy, is characterized by acute kidney injury and red blood cell (RBC) tubular casts in the kidney. 5/6 nephrectomy (5/7NE) rats treated with warfarin or dabigatran show changes in kidney function and morphology that are similar to human disease. The role of glomerular filtration rate (GFR) in the pathogenesis of ARN is not clear. The aim of these studies was to elucidate the role of GFR in the pathogenesis of dabigatran‐induced ARN in 5/6NE rats.

**Methods:**

5/6NE rats were treated per os with 150 mg/kg/day dabigatran alone or with drugs that lower (enalapril, 1.5 mg/kg/day) or increase (albuterol, 4.0 mg/kg/day) GFR for 7 days. Changes in coagulation and kidney function were recorded daily. Kidney morphology was evaluated on day 7 after the treatment.

**Results:**

Dabigatran resulted in activated partial thromboplastin time increase that was not affected by GFR‐modifying drugs. Blood pressure was significantly lower in 5/6NE rats treated with enalapril and dabigatran as compared to dabigatran alone. The GFR was decreased by 35% in enalapril/dabigatran‐ and increased by 26% in albuterol/dabigatran‐treated animals. There were no changes in serum creatinine, hematuria or urinary kidney injury molecule (KIM‐1) levels when GFR‐modifying drugs were added to dabigatran. All dabigatran‐treated animals had RBC casts in the kidney regardless of the GFR modification.

**Conclusions:**

GFR does not play a significant role in the dabigatran‐induced acute kidney injury in 5/6 nephrectomy model in rats. Based in these data, modification of GFR in patients with ARN is not warranted.

## Graphical abstract

Changes in the glomerular filtration rate (GFR) do not play a significant role in the development of anticoagulant‐related nephropathy (ARN), at least in the 5/6 nephrectomy rat model. Therefore, treatment with GFR‐modifying drugs does not appear to be beneficial for patients with ARN.

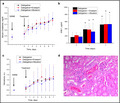

## 1 INTRODUCTION

Anticoagulant‐related nephropathy (ARN) has been described over 10 years ago as a complication of anticoagulation therapy first with warfarin and later with dabigatran and other anticoagulants (Brodsky et al., 2009, 2019). An animal model that shows similar kidney funcion changes and morphologic findings as in human ARN was developed (Ozcan et al., 2012; Ware et al., 2011). Our clinical observations and data from experimental animals indicate that ARN does not occur in individuals or animals with normal kidney function, even with excessive anticoagulation in humans or fatal anticoagulation in rats (Ryan et al., 2014; Ware et al., 2011; Ware, Feinstein, et al., 2015). Based on our constantly gaining experience with renal biopsy cases of ARN in humans, different underlying kidney diseases are seen in patients who developed ARN. They include focal segmental glomerular sclerosis (FSGS), diabetic nephropathy, mild immune complex depositions in the glomeruli or glomerular basement membrane abnormalities (Brodsky et al., 2009, 2019). Broad pathologic mechanisms lead to these diseases, but glomerular hyperfiltration/hyperperfusion is seen in many forms of chronic kidney disease, including secondary FSGS, hypertensive nephrosclerosis, and diabetic nephropathy (Apakkan Aksun et al., 2003; Nyberg et al., 1994; Reiser et al., 2014; Savin et al., 2003; Srivastava, 2006). Any injury to the kidney that results in a significant nephron loss may lead to glomerular hyperfiltration/hyperperfusion in the remaining nephrons.

Regulation of the glomerular filtration rate (GFR) includes many factors, and one of those are glomerular hemodynamics changes. Glomerular hemodynamics depends on the systemic blood pressure, distribution of the intrarenal blood flow, and contraction of the afferent and efferent arterioles (Brenner et al., 1976). Increased systemic blood pressure accelerates the progression of glomerulosclerosis in 5/6 nephrectomy rats and mice (Bidani et al., 1994). Our data indicate that anticoagulants, including warfarin and dabigatran, increase systolic blood pressure in both control and 5/6 nephrectomy rats (Ware, Vance, et al., 2015), which may participate in the ARN development. Treatments with ACE inhibitors or angiotensin receptor blockers provide renoprotection in 5/6 nephrectomy rats (Goncalves et al., 2004; Meyer et al., 1987; Vavrinec et al., 2012). ARN with warfarin or dabigatrancan be induced in 5/6 nephrectomy rats and it may be seen as early as 3 weeks after the ablative surgery (Ozcan et al., 2012; Ryan et al., 2014). At this stage, FSGS has not developed yet, but there is glomerular hyperfiltration/hyperperfusion (Hostetter et al., 1981; Meyer et al., 1987). There are several drugs in use in clinical practice that change the GFR, such as drugs that decrease intraglomerular pressure (ACE inhibitors, including enalapril (Major et al., 2008)) and drugs that increase glomerular filtration (β‐2‐adrenergic receptor agonists, such as albuterol (Jelkmann & Bauer, 1980)).

The aim of these studies was to investigate whether the modifications of the GFR affect the development of ARN secondary to dabigatran in 5/6 nephrectomy rats.

## 2 MATERIALS AND METHODS

These studies were approved by the Institutional Animal Care and Use Committees (IACUC) at Ohio State University.

Sprague Dawley male rats were obtained from the Charles River Laboratories. 5/6 nephrectomy was performed in 120–130 g rats as we described previously (Ware et al., 2011). Briefly, rats were anesthetized with isoflurane/oxygen (1:5), a middle laparotomy was performed, the right kidney was removed, as well as 2/3 of the left kidney. Hemostasis was achieved by hemostatic sponges (Quick clot; Z‐Medica Corporation). The incision was closed with 4.0 proline and the animals were kept at 12 hr/12 hr light/dark cycle on the standard rodent diet with free access to water.

Three weeks later, treatment with dabigatran etexilate (Boehringer Ingelheim Pharmaceuticals, Inc.) alone (150 mg/kg/day), dabigatran (150 mg/kg/day) and Enalapril (1.5 mg/kg/day), and dabigatran (150 mg/kg/day) and Albuterol (4.0 mg/kg/day) was begun. Drug dosage was determined based on our earlier studies and literature data (Brodsky et al., 1998; Lakhlani et al., 1994; Ryan et al., 2014; Slomowitz et al., 1999; Wang & Brooks, 1992).

All drugs were administered per os via gavage after blood pressure was measured and blood and urine samples were collected to reduce the harmful effects of the handling stress. Daily blood and urine samples were collected. GFR was measured on day 7 of the treatments by the inulin clearance, as we described earlier (Brodsky et al., 1998). Briefly, animals were anesthetized with isoflurane, placed on the heating table and p50 catheters were inserted in the left femoral vein and left femoral artery. The urinary bladder was catheterized with a p50 catheter as well. A 2% inulin solution dissolved in sodium saline (0.9% NaCl) was used. A bolus 0.5 ml infusion was made and the animal was constantly perfused thereafter via the femoral vein catheter at a rate of 1.5 ml/hr. After a 1 hr equilibration period, urine was collected from the urinary bladder for 60 min and a blood sample was collected from the femoral artery catheter into a heparinized vial. The blood was centrifuged for 10 min at 1,000 ***g*** and the inulin concentration was measured in the plasma and the urine by a colormetric method with Anthrone reagent. Serum and urine (diluted 1:10) were added to 3% trichloroacetic acid (10 mcl of serum/diluted urine to 90 mcl of 3% trichloroacetic acid (TCA)), 10 mcl of diluted samples were incubated with 250 mcl of Anthrone reagent for 25 min at 47°C and the absorbance was read at 620 nm by a Molecular Devices Versa Max plate reader (Molecular Devices) (Young & Raisz, 1952).

After measuring the GFR, the animals were sacrificed and the remnant kidney was dissected for histologic studies. Histology of the kidney was evaluated on 2–3 mcm sections of paraffin‐embedded tissue stained with hematoxylin and eosin (H&E).

Serum creatinine was measured based on the Jaffe reaction by a creatinine reagent assay (Pointe Scientific, Inc.) as we described earlier (Hostetter et al., 1981; Ware et al., 2011). Briefly, 10 μl of serum was mixed with 200 μl of working reagent at 37°C in a 96‐well plate and the absorbance was read at 510 nm at 40 and 100 s using the Molecular Devices Versa Max plate reader (Molecular Devices).

Hematuria was evaluated by dipsticks (Siemens reagent strips) and expressed in a semiquantitative scale from 0 to 3, where 0 is absent, 1 is mild, 2 is moderate, and 3 is severe.

Activated partial thromboplastin time (aPTT) was measured using a Fisher Scientific ThromboScreen 200 Hemostasis Analyzer (Fisher Scientific) based on the manufacturer protocol. Briefly, blood was collected into a tube containing 3.8% sodium citrate in a ratio of 9:1. The blood was centrifuged at 3,500 rpm for 10 min. Twenty microliters of plasma were placed in the incubation station with 20 μl of the aPTT reagent (Fisher Scientific). Then, after 3 min, preheated (at 37°C for 10 min) 20 μl of 0.025‐M calcium chloride was added. Clotting time was recorded in seconds.

Blood pressure was measured by a tail cuff using a blood pressure monitor (IITC Life Sciences Inc.), as we previously described (Ware, Vance, et al., 2015). The systolic, diastolic, and mean blood pressures were determined using the Blood Pressure Data Acquisition Software (IITC Life Sciences Inc. Version 1.35).

Urine kidney injury molecule 1 (KIM‐1) levels were determined by a quantikine immunoassay ELISA kit (R&D Systems) based on the manufacturer protocol. Briefly, urine was diluted 1:2, 50 mcl of assay diluent RD1W was added to the microplate well followed by 50 mcl of a sample or standard and incubated at room temperature for 2 hr. Each well was washed five times with wash buffer, 100 mcl of rat KIM‐1 conjugate was added to each well, and incubated for 2 hr at room temperature. Each well was washed five times with wash buffer, 100 mcl of substrate solution was added to each well, and incubated for 30 min at room temperature. Then, 100 mcl of stop solution was added to each well and the optical density of each well was determined at 540 and 450 nm. The reading at 540 nm was subtracted from the reading at 450 nm and the KIM‐1 concentration was calculated based on the standard curve.

### 2.1 Statistical analysis:

Descriptive statistics were used to analyze differences between experimental groups. Data are present as mean ± standard deviation (SD), unless otherwise specified. Student two‐tailed *t* test was used to analyze differences between two different time points within the same treatment group; one‐way ANOVA was used to analyze dynamic changes associated with the treatment within the same group and two‐way ANOVA was used to analyze differences between different treatment groups.

## 3 RESULTS

### 3.1 Anticoagulation, blood pressure, and glomerular filtration rate effects of dabigatran, enalapril, and albuterol

**FIGURE 1:**
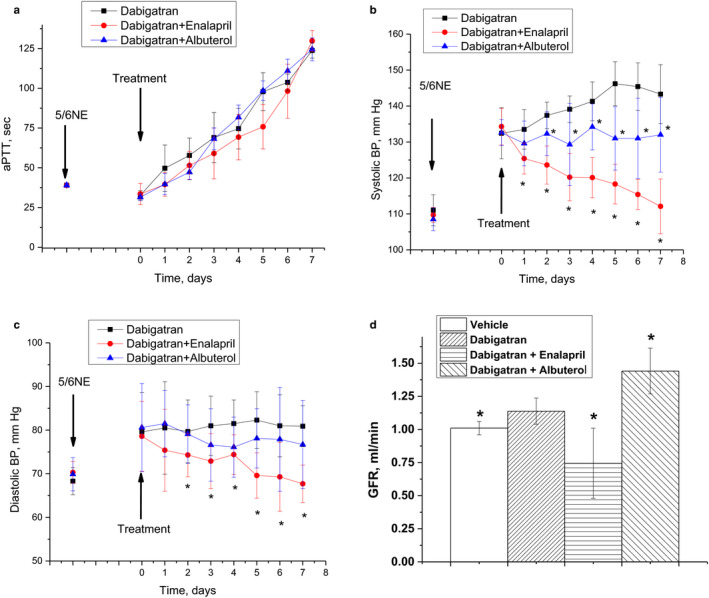
Effects of dabigatran and glomerular filtration rate (GFR)‐modifying drugs on anticoagulation, blood pressure, and GFR in 5/6 nephrectomy rats. (a) changes in activated partial thromboplastin time (aPTT) in 5/6 nephrectomy rats treated with 150 mg/kg/day dabigatran alone (*n* = 6) and with combination with enalapril (1.5 mg/kg/day) (*n* = 7) or albuterol (4.0 mg/kg/day) (*n* = 6). (b) changes in systolic blood pressure in 5/6 nephrectomy rats treated with 150 mg/kg/day dabigatran alone (*n* = 13) and with combination with enalapril (1.5 mg/kg/day) (*n* = 12) or albuterol (4 mg/kg/day) (*n* = 13). *p < .05 as compared to baseline (day 0 of the treatment). (c) changes in diastolic blood pressure in 5/6 nephrectomy rats treated with 150 mg/kg/day dabigatran alone (*n* = 13) and with combination with enalapril (1.5 mg/kg/day) (*n* = 12) or albuterol (4 mg/kg/day) (*n* = 13). *p < .05 as compared to baseline (day 0 of the treatment). (d) changes in the GFR in 5/6 nephrectomy rats treated for 7 days with 150 mg/kg/day dabigatran alone (*n* = 6) and with enalapril (1.5 mg/kg/day)/dabigatran (*n* = 5) or albuterol (4 mg/kg/day)/dabigatran (*n* = 5) or vehicle (*n* = 6). *p < .05 as compared to dabigatran alone. Data prior to the ablative surgery (5/6 NE) and treatment (arrows) are shown in a–c

Treatment with 150 mg/kg/day dabigatran resulted in a rapid increase in aPTT, similar to the data that we described earlier. Thus, by day 7 of treatment, aPTT was increased 4–5 folds from the baseline. Treatment with enalapril or albuterol did not change the anticoagulation effects of dabigatran (Figure 1a). Dabigatran alone increased systolic but not diastolic blood pressure; these effects were similar to those that we described earlier (Ware, Vance, et al., 2015). Treatment with enalapril/dabigatran resulted in the reduction of both systolic and diastolic blood pressures not only compared to dabigatran alone, but to the baseline as well (Figure 1b,c). Albuterol prevented dabigatran‐induced increase in the systolic blood pressure but did not change neither systolic nor diastolic blood pressures from the baseline (Figure 1b,c).

GFR was significantly modified with enalapril/dabigatran and albuterol/dabigatran treatments. Thus, animals treated with dabigatran and enalapril had a 35% decrease in GFR as compared to animals treated with dabigatran alone (0.74 ± 0.27 ml/min vs. 1.14 ± 0.10 ml/min, *p* = .0080). Animals treated with albuterol and dabigatran had GFR 26% higher than animals treated with dabigatran alone (1.44 ± 0.17 ml/min vs. 1.14 ± 0.10 ml/min, *p* = .0051). Dabigatran alone slightly increased the GFR (1.14 ± 0.10 ml/min vs. 1.01 ± 0.05 ml/min in vehicle‐treated 5/6 nephrectomy rats, *p* = .022), Figure 1d.

### 3.2 Glomerular filtration rate modification, anticoagulation, and kidney function

**FIGURE 2:**
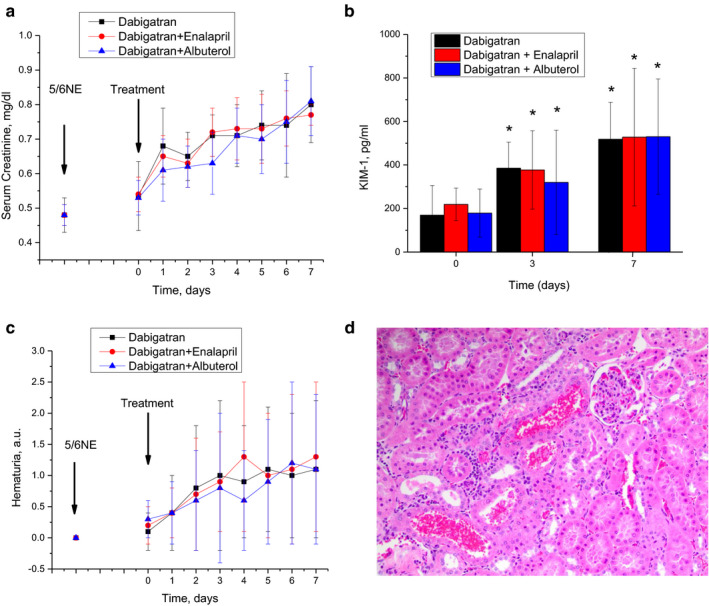
Effects of dabigatran and glomerular filtration rate (GFR)‐modifying drugs on kidney function and morphology. (a) changes in serum creatinine in 5/6 nephrectomy rats treated with 150 mg/kg/day dabigatran alone (*n* = 13) and with combination with enalapril (1.5 mg/kg/day) (*n* = 12) or albuterol (4 mg/kg/day) (*n* = 13). (b) changes in urine kidney injury molecule 1 (KIM‐1) in 5/6 nephrectomy rats treated with 150 mg/kg/day dabigatran alone (*n* = 8) and with combination with enalapril (1.5 mg/kg/day) (*n* = 8) or albuterol (4 mg/kg/day) (*n* = 8). *p < .05 as compared to baseline (day 0 of the treatment). (c) changes in hematuria in 5/6 nephrectomy rats treated with 150 mg/kg/day dabigatran alone (*n* = 13) and with combination with enalapril (1.5 mg/kg/day) (*n* = 12) or albuterol (4 mg/kg/day) (*n* = 13). (d) a representative image of the kidney from a 5/6 nephrectomy rat treated with 150 mg/kg/day dabigatran and 1.5 mg/kg/day enalapril for 7 days. Similar morphology (red blood cell casts) was seen in other treatment groups as well. Hematoxylin‐eosin stain, magnification ×200. Data prior to the ablative surgery (5/6 NE) and treatment (arrows) are shown in a–c

Treatment with 150 mg/kg/day dabigatran resulted in a serum creatinine increase, similar to the data that we described earlier (Ryan et al., 2014). Thus, serum creatinine increased from 0.54 ± 0.10 to 0.80 ± 0.11 mg/dl by day 7 after the treatment (*p* < .0001). Neither 1.5 mg/kg/day enalapril nor 4.0 mg/kg/day albuterol changed this dabigatran‐induced increase in serum creatinine (enalapril and dabigatran‐treated 5/6 nephrectomy rats had serum creatinine increase from 0.55 ± 0.06 to 0.78 ± 0.03 mg/dl by day 7, *p* < .0001; albuterol and dabigatran‐treated 5/6 nephrectomy rats had serum creatinine increase from 0.53 ± 0.06 to 0.81 ± 0.11 mg/dl, *p* < .0001), Figure 2a.

Acute kidney injury was evident not only by the serum creatinine increase, but by the excretion of KIM‐1 in the urine as well. Rats treated with 150 mg/kg/day dabigatran had an increase in urinary KIM‐1 from 169.5 ± 138.6 to 384.9 ± 119.9 pg/ml by day 3 and to 517.8 ± 169.3 pg/ml by day 7, *p* = .003. This trend was not changed by GFR‐modifying drugs (*p* = .9698). Enalapril and dabigatran‐treated 5/6 nephrectomy rats had a KIM‐1 increase in the urine from 218.8 ± 74.8 to 377.3 ± 179.4 pg/ml by day 3 and to 528.1 ± 316.1 pg/ml by day 7, *p* = .0298. Albuterol and dabigatran‐treated 5/6 nephrectomy rats had KIM‐1 increase in the urine from 178.9 ± 110.6 to 319.7 ± 240.7 pg/ml by day 3 and to 531.0 ± 265.2 pg/ml by day 7, *p* = .0131, Figure 2b.

Similar to serum creatinine changes, 150 mg/kg/day dabigatran increased hematuria from 0.13 ± 0.3 to 1.1 ± 1.1 a.u. by day 7, *p* = .0093. GFR‐modifying drugs did not change this dabigatran‐associated increase in hematuria, *p* = .9932, Figure 2c. Enalapril and dabigatran‐treated 5/6 nephrectomy rats had hematuria increase from 0.17 ± 0.32 to 1.32 ± 1.2 a.u. by day 7, *p* = .0045; albuterol and dabigatran‐treated 5/6 nephrectomy rats had hematuria increase from 0.27 ± 0.33 to 1.1 ± 1.1 a.u., *p* = .0253).

Morphologically, all 5/6 nephrectomy rats treated with dabigatran had acute tubular epithelial cell injury and red blood cell (RBC) casts in the tubules, regardless of a GFR‐modifying drug (Figure 2d).

## 4 DISCUSSION

Our studies are the first to investigate the role of GFR in the pathogenesis of ARN. Our clinical experience with patients who developed unexplained acute kidney injury while on anticoagulation therapy had demonstrated that all these patients had an underlying glomerular disease (Brodsky et al., 2009, 2019). Regardless of the etiology of chronic kidney injury, with progressing nephron loss, there is an increase in the glomerular perfusion/filtration in the remaining nephrons.

Hypertension accelerates the progression of FSGS in 5/6 nephrectomy rats (Bidani et al., 1994). In our human studies, we found that patients with warfarin‐related nephropathy (WRN) had hypertension more often than those who did not develop WRN. Also, there were more patients who were treated with drugs that raise glomerular hydrostatic pressure (e.g., dihydropyridine calcium‐channel blockers, direct‐acting smooth muscle relaxants, potassium channel agonists, b‐2‐adrenergic receptor agonist, erythropoietin, and endothelin receptor antagonist) among those who developed WRN as compared to the non‐WRN group. However, the percentage of patients who were on medications that lower glomerular hydrostatic pressure (e.g., ACE inhibitors, angiotensin receptor blockers, b‐blockers, diuretics, non‐dihydropyridine CCB, clonidine) was also higher in WRN than in the non‐WRN group (Brodsky et al., 2011). Based on the outcome of the current studies, this paradox could be explained by the fact that the modification of the GFR does not affect the development of ARN and there are other pathogenetic mechanisms that allow RBC passage through the glomerular filtration barrier.

It is difficult to study the permeability of the glomerular filtration barrier to RBC. Mainly, such studies involve in vivo multiphoton microscopy that investigates the permeability to different fluorescent‐labeled sugars or proteins. It has been demonstrated that increased glomerular filtration barrier permeability to high‐molecular weight dextran is restricted to the areas of podocyte injury (Peti‐Peterdi & Sipos, 2010). This mechanism may be different for RBC. In many human glomerular diseases (such as IgA nephropathy, thin basement membrane nephropathy, etc), there is no obvious podocyte injury by ultrastructural examination, but these diseases are characterized by hematuria. Among our patients with ARN, the most common morphologic finding was mild immune complex deposition in the glomeruli (53% of patients). Pauci‐immune crescentic necrotizing glomerulonephritis was seen in 20% of patients. Only 10% of patients had FSGS and 5% of patients had diabetic glomerulosclerosis (Brodsky et al., 2019). It appears that only two latter kidney diseases had some changes in the GFR, whereas the majority of patients with ARN had a glomerular injury that is related either to immune complexes or other etiologies (ANCA‐associated pauci‐immune crescentic glomerulonephritis).

Among the limitations of this study is that only anticoagulation with thrombin inhibitor (dabigatran) was investigated. However, we demonstrated that the effects of dabigatran on the kidney in regards to the ARN development in rats are similar to those of warfarin (Ryan et al., 2014). Also, dabigatran‐associated ARN currently is the most commonly reported after warfarin‐induced ARN in humans, indicating the importance to investigate anticoagulation with dabigatran (Brodsky et al., 2019). With the development of direct oral anticoagulants (one of those is dabigatran), the proportion of warfarin‐treated patients is decreasing (Wu et al., 2020). Other direct anticoagulants (such as apixaban) appear to have lessened frequency in the ARN development, since only a few cases were reported (Brodsky et al., 2017).

In conclusion, our data indicate that changes in the GFR do not play a significant role in the development of ARN, at least in the 5/6 nephrectomy rat model. Therefore, treatment with GFR‐modifying drugs does not appear to be beneficial for the patients with ARN.
